# The challenging diagnosis of ICU-related Mesenteric Ischaemia: a prospective, observational, multicentre cohort

**DOI:** 10.1016/j.aicoj.2026.100028

**Published:** 2026-01-21

**Authors:** Stefan Andrei, Jerome Allyn, Nicolas Allou, Sonia Yung, Gabriel Stefan, Peter Matthews, Mathieu Desmard, Laura Federici, Yves Castier, Lara Ribeiro Parenti, Philippe Montravers, Pascal Augustin

**Affiliations:** a Department of Anesthesiology and Intensive Care, AP-HP Nord, Bichat-Claude Bernard University Hospital, Paris, France; b Group of Data Modeling, Computational Biology, and Predictive Medicine, Applied Mathematics, CNRS UMR 81987, INSERM U1024, IBENS, Ecole Normale Supérieure, Paris, France; c Intensive Care Unit, Saint-Denis University Hospital, La Reunion, France; d Center for Clinical Investigation (CIC) 1410 Clinical Epidemiology National Institute of Health and Medical Research (INSERM), Reunion University Hospital, Saint Pierre, Reunion Island, France; e Department of Nephrology, Dr Carol Davila Teaching Hospital of Nephrology, University of Medicine and Pharmacy Carol Davila, Bucharest, Romania; f Department of Emergency Medicine, Princess Alexandra Hospital, Harlow, United Kingdom; g Medicosurgical Intensive Care Unit, Institut Médico-chirurgicale Montsouris, Paris, France; h Medicosurgical Intensive Care Unit, Sud Francilien Hospital, Corbeil Essones, France; i Department of Thoracic and Vascular Surgery, AP-HP Nord, Bichat-Claude Bernard University Hospital, Paris, France; j Department of General Surgery, AP-HP Nord, Bichat-Claude Bernard University Hospital, Paris, France; k Inserm UMRS 1149, Centre de Recherche sur l'Inflammation, Université Paris Cité, Paris, France; l University of Paris, Physiopathology and Epidemiology of Respiratory Diseases, INSERM UMR1152, Paris, France

**Keywords:** Necrotic bowel, ICU, Diagnosis, Acute mesenteric ischemia

## Abstract

**Background:**

The diagnosis of acute mesenteric ischemia (AMI) is challenging, especially in the intensive care unit (ICU), where non-occlusive mesenteric ischemia (NOMI) predominates. In ICU patients, contrast-enhanced computed tomography (CT) provides limited diagnostic accuracy, and no single biomarker is sufficiently reliable. Transmural digestive necrosis, revealed as necrotic bowel (NB) during surgical exploration, is irreversible and requires bowel resection. We proposed an approach combining several clinical, biological, and therapeutic parameters to predict the presence of NB in ICU patients with high suspicion of AMI.

**Methods:**

We conducted a prospective observational study in three ICUs. All consecutive patients with suspected AMI were enrolled. Patients with NB identified during surgical exploration were compared with those without NB. Patients who survived without undergoing surgery were considered not to have NB. Multivariable logistic regression analysis was used to identify parameters independently associated with NB.

**Results:**

A total of 202 patients were included. Among them, 74 (37%) had NB (including 70 with NOMI and 4 with occlusive AMI), while 128 (63%) did not. In the multivariable analysis, age (OR 1.068, 95% CI 1.027–1.111, p = 0.001), active fluid removal (OR 3.148 (1.19−8.33), p = 0.021), signs of gastrointestinal injury (3.432 (1.082−10.885), p = 0.036), need for renal replacement therapy (OR 3.834 (1.457−10.01), p = 0.006), and lactate dehydrogenase (log) at the time of AMI suspicion (OR 7.135 (2.1−24.235) p = 0.002) were independently associated with NB. Among biomarkers, lactate dehydrogenase, showed the highest area under the ROC curve.

**Conclusions:**

This is the first study to propose a combined approach for predicting NB in ICU patients with suspected AMI. When AMI is highly suspected, surgical exploration should be considered in patients presenting with signs of gastrointestinal injury in a context of fluid removal or renal replacement therapy, as these findings are strongly suggestive of necrotic bowel.

## Background

Acute Mesenteric Ischaemia (AMI) is an underdiagnosed cause of acute abdomen in the intensive care unit (ICU) [1,2]. Non-occlusive mesenteric ischaemia (NOMI) primarily affects critically ill patients. Contrary to occlusive MI, NOMI is due to reduced systemic blood flow and vasoconstriction in the splanchnic territories [3]. Histological features of AMI range from the less severe, reversible mucosal ischaemia, to the final, irreversible, stage of transmural digestive necrosis. At this stage, the surgical exploration discloses a necrotic bowel (NB), and the only curative treatment is bowel resection [4].

Risk factors for AMI in the ICU setting are multifaceted, including cardiovascular conditions such as shock, use of vasopressors, open/endovascular aortic procedures, or post-cardiopulmonary bypass surgery [[5], [6], [7]]. Of note, AMI after aortic surgery is classified as NOMI. Other common conditions in ICU patients, such as chronic kidney disease, hemodialysis, severe burns, or acute pancreatitis, are predisposing conditions for AMI [5,8,9].

Due to the high mortality rate of AMI, timely diagnosis and management are of crucial importance for these already severely ill patients [1,2,10]. The diagnosis in the ICU remains challenging, because AMI occurs in patients already hospitalized for a preexisting pathology. The suspicion of AMI may also be delayed because of the difficult clinical assessment of intubated and sedated patients. The concept of gastrointestinal (GI) dysfunction has been proposed [11], to fill the lack of GI component in the different severity scoring systems in ICU patients, and it may help in the diagnosis of abdominal conditions in the ICU setting. While contrast-enhanced CT imaging is the cornerstone of occlusive AMI diagnosis, the performance of CT imaging is still insufficient to diagnose NOMI [[12], [13], [14]]. Medico-surgical team would benefit from having effective decision-making tools for performing exploratory surgery. In the ICU setting, the decision-making process of this emergent surgical intervention takes into account both risks: on one hand, the risk of unnecessary transfer in the operating room [15] and undue surgical exploration in severely-ill patients, and on the other hand, the risk of delaying necessary bowel resection.

Different attempts have been made to improve diagnosis of AMI, with promising preliminary results, such as for D-lactate, citrulline, and intestinal fatty acid binding protein (IFABP) [16]. Unfortunately, those biological tests are not routinely available, and they have finally failed to prove to be accurate enough as single isolated biomarkers [[17], [18], [19]].

We hypothesized that a multiple-criteria diagnostic strategy may be superior to a single biomarker approach. Thus, the primary aim was to determine routine clinical and biological markers associated with NB compared to patients without NB.

## Methods

### Ethics

The study received the ethical approval from an independent ethical committee (CERAR: IRB 00010254 - 2016 – 085), registered at la CNIL (commission nationale informatique et libertés). The study protocol was conducted in accordance with the Declaration of Helsinki from 1968. The patients or their next of kin were informed of the study. The study was purely observational and used existing, routinely collected data. Therefore, written informed consent was not required, in compliance with the French law (loi Jardé n° 2012–300). The manuscript was drafted based on STROBE guidelines [20].

### Patients management and study design

This was a prospective, observational study in three ICUs, over a 4-year period.

We included adult patients with a high clinical suspicion of AMI for the ICU team/treating intensivist, while already hospitalized in ICU for any other reason.

High clinical suspicion of AMI was defined by: i) the presence of signs of gastrointestinal injury (abdominal rigidity or guarding, abdominal pain when clinically examinable, enteral feeding intolerance, abdominal distension, absence of bowel sounds, paralytic ileus, melena, or new-onset diarrhea); and/or ii) the new onset/worsening organ dysfunction, including acute hemodynamic failure, without an alternative explanation. The high clinical suspicion of AMI needed to prompt at least one of the following diagnostic procedures: contrast-enhanced abdominal CT, digestive endoscopy, or surgical exploration (laparotomy/laparoscopy). Clinical features leading to high clinical suspicion for AMI are given in supplemental data (Table S1). Patients were included prior to the results of these investigations. We did not include patients with AMI features unexpectedly discovered on a CT scan, digestive endoscopy, or surgical exploration performed for other reasons. All types of bowel ischaemia, including colonic ischaemia were considered.

The exclusion criteria were: (1) age <18 years, (2) ongoing pregnancy. Patients with uncertain definitive diagnosis regarding the presence or absence of NB were not included in the study analysis.

Because it was an observational study, the diagnostic process and the management of patients were not protocolized.

All patients with NB witnessed at surgical exploration, underwent a segmental bowel resection, except when considered futile due to ethical considerations.

### Patients' classification and definitions

”Necrotic bowel” was an intraoperative observation made by the surgeon.

Two groups were defined: the” NB group” and the” No NB group”.

The” NB group” comprised patients with intraoperative visualisation of a non-viable segment of necrosis [19].

The” No NB group” comprised patients with no NB observed on surgical exploration (with or without mucosal AMI), or patients who survived without surgical exploration, who were considered not to have NB, as previously described [4].

Patients who did not receive surgical exploration were classified as follows: In the” no NB group" if they survived; or as having an uncertain diagnosis if they died.

New-onset or worsening of organ dysfunction: as defined by organ-specific SOFA score [21].

Need for RRT: Initiation of RRT was not protocolized but decided on an individual basis, according to current practice [22].

”Signs of GI injury” was a composite variable that included: abdominal rigidity or guarding, abdominal pain, enteral feeding intolerance, abdominal distension, absence of bowel sounds, paralytic ileus, melena and new-onset diarrhea [23].

Chronic kidney disease was assessed by the preadmission estimated glomerular filtration rate below 60 ml/min as calculated by the chronic kidney disease epidemiology collaboration (CKD-EPI) creatinine equation [24].

Active fluid removal: negative fluid balance achieved with diuretics and/or RRT [25].

### Data collection

Patients’ demographic characteristics were collected: age, weight, height, admission sequential organ failure assessment score (SOFA) [21], chronic medications, comorbidities, cardiovascular diseases, including permanent or paroxysmal atrial fibrillation. The ICU medical context was recorded: initial reason for ICU admission, type of admission (post general surgery, post-aortic procedures, post-cardiac procedures, or medical admission), and the following significant events within the 3 days preceding the clinical suspicion of AMI: active fluid removal (using diuretics or RRT), or need for catecholamines. Histopathologic analyses of the resected bowel were not collected.

The clinical features leading to a high clinical suspicion of AMI were recorded. Patients’ clinical and laboratory characteristics at the time of AMI suspicion were recorded: ongoing continuous sedation, SOFA score, need for catecholamines, systolic blood pressure, acute organ dysfunctions defined from SOFA score; presence of new onset or worsening of organ dysfunctions, including need for RRT. Signs of GI injury and the presence of any area of skin mottling were also recorded. Blood analyses at the time of clinical suspicion of AMI were also collected: arterial blood pH, arterial lactate level, liver enzymes, bilirubin, lactate dehydrogenase (LDH), creatine kinase (CK), white blood cells, and platelet count. The final diagnosis regarding the presence of NB witnessed intraoperatively, and the patients’ ICU outcomes (death in ICU and length of stay in ICU) were also collected.

### Study endpoint

The study endpoint was the diagnosis of NB, documented intraoperatively.

### Statistical analyses

All analyses were performed using R software version 3.4.4 (R Foundation for Statistical Computing, Wien, Austria). A bilateral p value <0.05 was considered to be statistically significant.

The quantitative variables were presented as mean (± standard deviation) or as median [25%; 75% interquartile range], as appropriate. The qualitative variables were presented as numbers (percentages). The normality of quantitative variables was verified using histograms and the Shapiro–Wilk test. Continuous variables were compared using the Student t-test or the Mann–Whitney test, as appropriate. Categorical variables were compared using the Chi-square test, and Fisher exact test. Because of the lack of literature on the subject of AMI in ICU, the a priori sample size calculation would be purely speculative [26].

To evaluate the variables associated with the diagnosis of NB, we used a combined statistical approach. First, we used a univariate logistic model with NB as the dependent variable and patients’ characteristics as independent variables. We further constructed a multivariable logistic model based on the clinically pertinent and statistically significant variables in univariate logistic analyses (p < 0.05). The conditions of validity for the multivariate logistic model were verified to have at least 10 events for each independent variable included in the model. Several multivariate models were performed and evaluated. Redundant variables were avoided based on the variance influence factor (VIF). A VIF > 2 was considered a relevant indicator of collinearity. The final model was selected based on the best Akaike Information Criterion (AIC). For scaling purposes, some quantitative variables were log-transformed.

The cutoffs of continuous variables (LDH and age) were determined by the Younden's index. Receiver operator-characteristic (ROC) curves were also performed to test the ability of candidate biomarkers to predict the presence of NB. The area under curves (AUC) were determined. PROC package [27] was used to assess the AUC.

## Results

### Baseline characteristics of the cohort

**Fig. 1:**
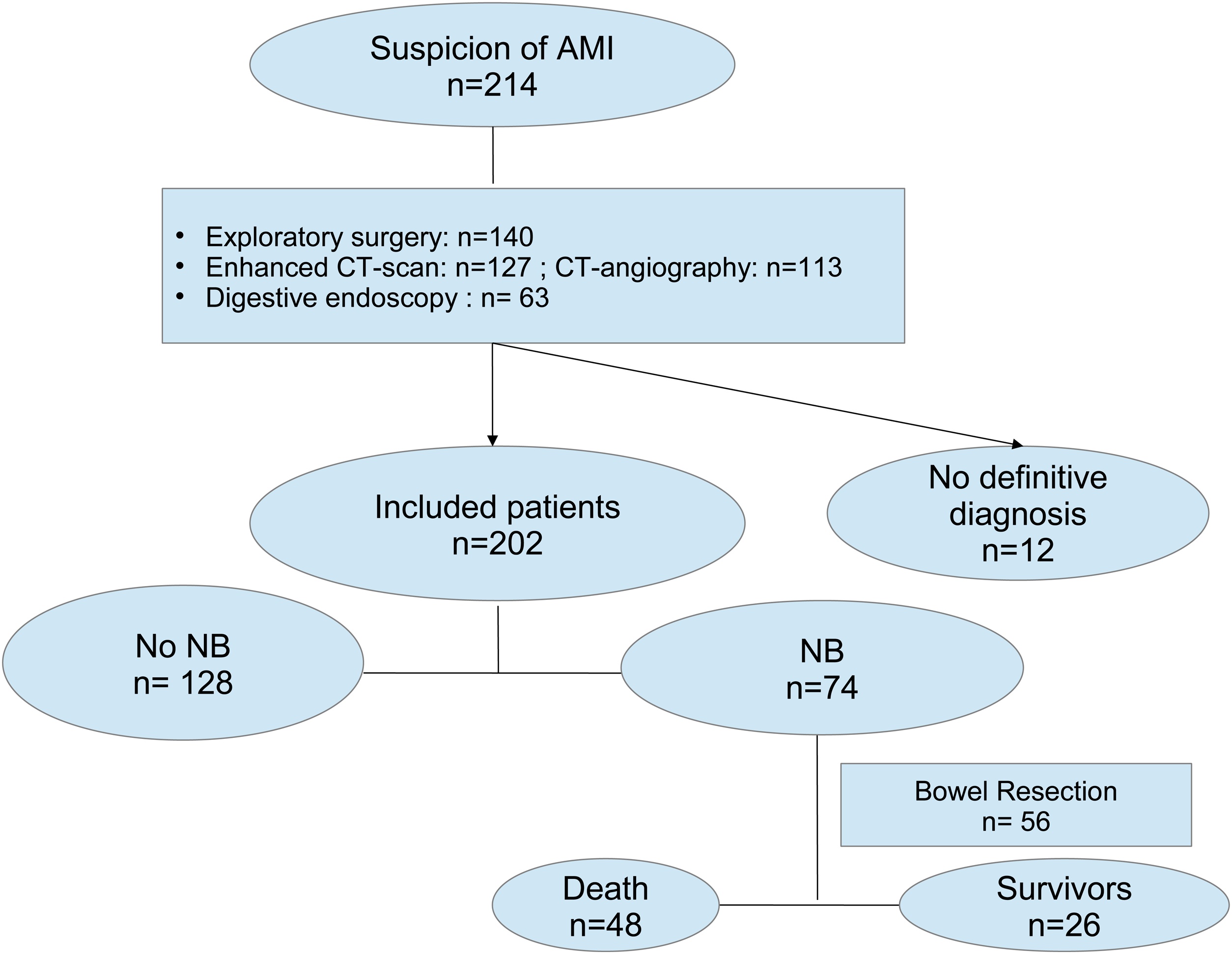
Flow chart of patients with AMI suspicion. Abbreviations: AMI: Acute Mesenteric Ischaemia; CT: computed Tomography; NB: Necrotic Bowel.

**Fig. 2:**
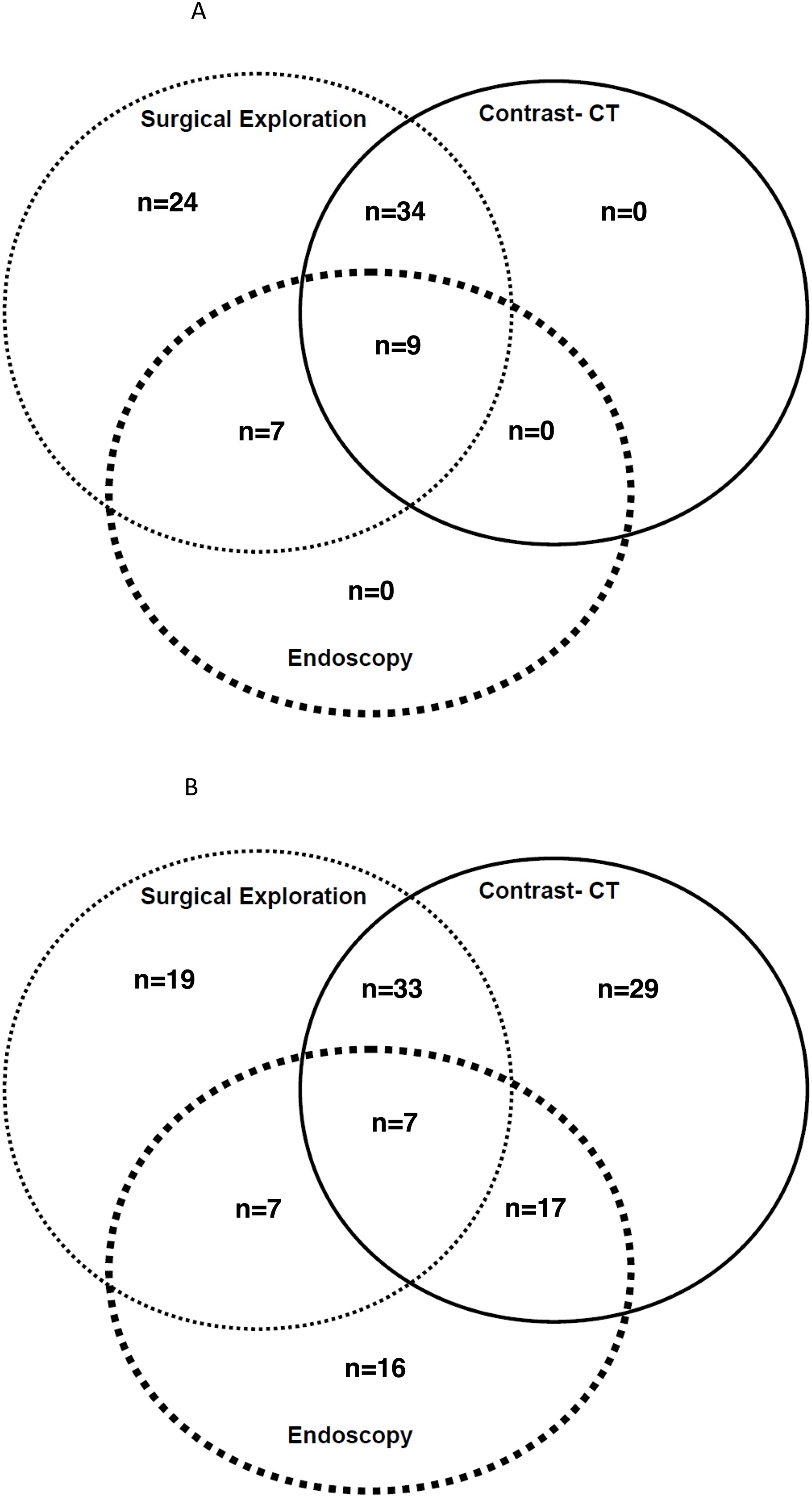
Distribution of diagnostic procedures for AMI suspicion.

A total of 214 patients were suspected of AMI in the ICU. Twelve had no definitive diagnosis. Finally, 202 patients remained in the main analysis (Fig. 1). Patients had been admitted for medical reasons, for perioperative complications, or for planned postoperative care. Seventy patients (35%) were admitted in the context of postoperative aortic surgery. At the time of suspicion, 99 (49%) patients were sedated. Clinical features leading to the high suspicion of AMI are given in supplemental data (Table S1). The numbers of diagnostic procedures are described in Fig. 2A and B, and their findings are shown in supplemental data (Table S2).

Seventy-four patients were found with NB (including 70 NOMI, 4 occlusive AMI, and none with venous AMI), and 128 with no NB. Outcome of patients according to their final diagnosis is given in Table 1. Patients’ baseline characteristics are displayed in Table 2. We noted that patients in the NB group were older (67 years old vs 63 years old, p = 0.008). There were no other differences, including pre-existing cardiovascular conditions.

**Table 1:** Outcome of patients with AMI suspicion.

	All patients (202)	No NB (128)	NB (74)
Death in ICU, n (%)	88 (44)	40(31)	48(65)
Length of stay in ICU* (days)	10 [4–20]	9 [4–20]	13[7–20]

Abbreviations: NB = Necrotic Bowel; ICU = Intensive Care Unit;

* For survivors only.

**Table 2:** Patients’ baseline characteristics.

Variables	All patients (202)	No NB (128)	NB (74)	p-value
Age (years)	64 [57;73]	63 [55;71]	67 [59;75]	0.008
Gender (female)	48 (28)	21 (29)	27 (21)	0.220
Hypertension	134 (66)	81 (63)	53 (72)	0.227
Type 2 diabetes mellitus	38 (19)	26 (20)	12 (16)	0.473
Insulin-treated diabetes mellitus	17 (9)	12 (9)	5 (7)	0.508
Active or past-history of smoking	109 (54)	70 (55)	39 (53)	0.785
Dyslipidemia	79 (39)	51 (40)	28 (38)	0.778
CKD	46 (23)	27 (21)	19 (26)	0.454
Chronic hemodialysis	16 (8)	9 (7)	7 (10)	0.522
Vascular artery disease	127 (63)	79 (62)	48 (65)	0.656
CAD	56 (28)	43 (34)	13 (18)	
Peripheral AD	98 (51)	60 (47)	38 (52)	
Antiplatelets treatment	117 (58)	78 (61)	39 (53)	0.253
Statins	102 (51)	64 (50)	38 (51)	0.853
Beta blockers	81 (40)	54 (42)	27 (37)	0.426
Calcium inhibitors	44 (22)	33 (26)	11 (15)	0.064
ACEI or ARB	64 (34)	39 (31)	29 (40)	0.223
AF (permanent or paroxysmal)	50 (25)	36 (28)	14 (19)	0.144

Abbreviations: NB = Necrotic Bowel; CKD = Chronic Kidney Disease; CAD = Coronary Artery Disease; SOFA = Sequential Organ Failure Assessment ACEI = Angiotensin Converting Enzyme Inhibitor; ARB = Angiotensin II Receptor Blocker; AF = Atrial Fibrillation.

### Characteristics upon suspicion of AMI

Characteristics upon ICU admission and potential predisposing events within the previous 72 h for AMI are displayed in Table 3. Patients of the NB group had a higher SOFA score on admission compared to others (10 ± 4 vs 8 ± 4, p = 0.009). The patients’ clinical and laboratory blood results at the time of AMI suspicion are shown in Table 4. Of note, arterial lactate concentrations were not statistically different (2.8 mmol/L for no NB group vs 3.4 mmol/L for the NB group, p = 0.289)

**Table 3:** Characteristics at ICU admission and prior to AMI suspicion.

Variables	All patients (202)	No NB (128)	NB (74)	p-value
Type of hospitalization in ICU				0.223
Medical	49	27(21)	22(30)	
Surgical	153	101(79)	52(79)	
SOFA upon admission	9 ± 4	8 ± 4	10 ± 4	0.009
Aortic surgery	70 (35)	49 (38)	21 (28)	0.154
Active fluid removal within the previous 72 h	35 (17)	16 (13)	19 (26)	0.028
Need for catecholamines within the previous 72 h	111 (55)	69 (54)	42 (57)	0.695

Abbreviations: NB = Necrotic Bowel; ICU = Intensive Care Unit; SOFA = Sequential Organ Failure Assesment; RRT = Renal Replacement Therapy.

**Table 4:** Patients’ characteristics at the time of mesenteric ischemia suspicion.

Variables	All patients (202)	No NB (128)	NB (74)	p-value
GI failure	156 (77)	91 (71)	65 (88)	0.006
Organ dyfunction	176 (87)	112 (88)	64 (87)	0.836
Respiratory dysfunction	101 (51)	61 (48)	40 (54)	0.441
Neurological	42 (21)	29 (23)	13 (18)	0.361
New-onset renal failure	138 (68)	81 (64)	57 (77)	0.019
De novo RRT	77 (38)	39 (30)	38 (51)	0.005
Need for catecholamines	149 (74)	91 (72)	58 (78)	0.294
Art. lact D1 (mmol/L)	3 [1.7−6.1]	2.8 [1.5−6]	3.4 [2−6.6]	0.289
Arterial pH	7.30 [7.18;7.40]	7.32 [7.21−7.41]	7.25 [7.17−7.37]	0.040
Alkaline reserve (mmol/L)	19 [15–22]	19 [16–24]	18 [14–21]	0.017
Creatin Kinase (IU/L)	400 [152−1860]	373 [143−1760]	442 [167−3460]	0.353
Bilirubin (µmol/L)	13 [9–22]	12 [9–20]	14 [10–27]	0.208
LDH (IU/L)	473 [276−848]	392 [255−784]	672 [425−1426]	<0.001
AST (IU/L)	83 [46−300]	79 [41−230]	109 [52−656]	0.020
ALT(IU/L)	50 [27−182]	48 [22−99]	73 [31−342]	0.022
PTap (%)	64 ± 23	66 ± 23	59 ± 21	0.025
Creatinin (µmol/L)	172 [112−273]	152 ]100−249]	200 [134−329]	0.01
Alkaline phosphatase	85 [53−130]	89 [57;161]	75 [56−111]	0.194
Hemoglobin (g/dl)	94 [84−113]	91 [80−107]	105 [88−160]	0.001
WBC (G/L)	13.4 [9–19]	13.6 [9.5−18.6]	13.0 [8.8−20.2]	0.882
Platelets (G/L)	167 [100−254]	174 [103−260]	164 [95−235]	0.355
Abnormal temperature*	76 (38)	47 (44)	29 (48)	0.548
SOFA suspicion	9 [5–12]	8 [4;11]	12 [8–14]	<0.001
Mottled skin	98 (49)	57 (45)	41 (55)	0.104
SAP lowest (mmHg)	80 [70−95]	81 [70−99]	80 [69−92]	0.329

Abbreviations: NB = Necrotic Bowel; GI = Gastrointestinal; RRT = Renal Replacement Therapy; Art.lact D1 = Total arterial lactate at Day 1 of suspicion; LDH = Lactate Dehydrogenase; AST = Aspartate Aminotransferase; ALT = Alanine Aminotransferase; PTap = Prothrombin time activity percentage; WBC = White Blood Cells; SOFA = Sequential Organ Failure Assessment; SAP = Systolic Arterial Pressure;

* Temperature <36 °C, or >38.5 °C.

Patient’s age, active fluid removal prior to AMI suspicion, need for RRT, signs of GI injury, the SOFA score at the time of suspicion, LDH concentration level, and arterial blood pH at the time of AMI suspicion were included in the final multivariable logistic regression model (Table 5). In the multivariable analysis, age (OR 1.068, 95% CI 1.027–1.111, p = 0.001), active fluid removal (OR 3.148 (1.19−8.33), p = 0.021), signs of gastrointestinal injury (3.432 (1.082−10.885), p = 0.036), need for renal replacement therapy (OR 3.834 (1.457−10.01), p = 0.006), and log (lactate dehydrogenase) at the time of AMI suspicion (OR 7.135 (2.1−24.235) p = 0.002) were associated with NB (Table 5).

**Table 5:** Multivariable logistic regression analysis with NB as dependent variable.

Variable	Estimate	OR (95% Confidence Interval)	p
Age	0.066	1.068 (1.027−1.111)	0.001
pH at suspicion (1 unit)	1.607	4.986 (0.219−113)	0.414
SOFA at suspicion (1 unit)	0.060	1.062 (0.948−1.189)	0.300
Signs of GI injury (yes vs no)	1.233	3.432 (1.082−10.885)	0.036
Active fluid removal (yes vs no)	1.147	3.148 (1.19−8.33)	0.021
De novo RRT (yes vs no)	1.344	3.834 (1.457−10.01)	0.006
LDH (log)	1.965	7.135 (2.1−24.235)	0.002

Abbreviations: NB = Necrotic Bowel; SOFA = Sequential Organ Failure Assessment; GI = Gastrointestinal; RRT = Renal Replacement Therapy; LDH = Lactate Dehydrogenase.

The analysis was repeated only in patients who had a surgical exploration. Multivariable analysis found the same variables associated with the diagnosis of NB except for LDH plasma concentration (Supplemental data, Table S3, S4, S5, S6).

### Performance of diagnostic criteria

Optimal cutoffs were 65 years old and 405 IU for patients' age and LDH, respectively, as determined by the Younden's index. Table 6 shows diagnostic performance for each of the 5 criteria. The cumulative performance of these criteria is given according to the number of criteria met in Table 7A and B.

**Table 6:** Performance of the 5 diagnostic criteria.

	Number of patients	Sensitivity	Specificity	Positive predictive value	Negative predictive Value
Age > 65 year-old	90	0.55	0.62	0.46	0.71
Active fluid removal	35	0.26	0.88	0.54	0.67
De novo RRT	77	0.51	0.7	0.49	0.71
Signs of GI injury	156	0.88	0.29	0.42	0.8
LDH > 405 IU	130	0.84	0.47	0.48	0.83

Abbreviations: RRT = Renal Replacement Therapy; GI = Gastrointestinal; LDH = Lactate Dehydrogenase.

**Table 7:** Diagnostic accuracy according to the number of criteria.

A			
Number of criteria	Number of patients	Number of patients with NB	Percentage of patients with NB
0	9	1	11%
1	27	2	7%
2	72	16	22%
3	59	29	49%
4	35	26	74%
5	0	–	–

B			
Number of criteria	Number of patients	Number of patients with NB	Percentage of patients with NB
≥0	202	74	37%
≥1	193	73	38%
≥2	166	71	43%
≥3	94	55	59%
≥4	35	26	74%
5	0	**–**	**–**

### Biomarkers predictive value

Some biomarker concentrations were significantly higher in patients with NB compared to others (supplement data, Table S7). However, their ability to predict NB was poor, and the highest AUC was observed with LDH: AUC 0.685, 95% CI (0.603−0.766), p < 0.001.

## Discussion

In the present study, we aimed to investigate the diagnostic criteria for NB in ICU patients suspected of AMI. Our main findings indicate that the need for RRT, older age, signs of GI injury, elevated LDH levels, and active fluid removal within the previous 72 h were all associated with NB. Although no single biomarker or clinical criterion alone provides satisfactory diagnostic performance, their combination improves diagnostic accuracy.

Many previous studies have relied on diagnostic strategies centered on CT imaging. Bourcier et al. reported a negative predictive value of only 48% in ICU patients with suspected NOMI [13], which does not support decision-making based solely on contrast-enhanced CT findings. Given the limited sensitivity of CT scans in NOMI, we proposed a decision-making strategy that does not rely primarily on CT imaging. Consequently, our study did not incorporate CT results.

The diagnosis of NB is often delayed in the ICU setting [1,[28], [29], [30]], and the relatively poor performance of CT scans does not allow reliable exclusion of NOMI [31].

Our findings suggest that, in cases of high clinical suspicion of AMI, the presence of signs of GI injury in the context of fluid removal or RRT should prompt consideration of surgical exploration to rule out NB, provided no alternative diagnosis is identified. Although age was statistically significant in the multivariable analysis, its clinical relevance as an isolated diagnostic criterion appears limited in daily practice.

These results regarding factors associated with NB were consistent with previous literature. The potential role of RRT and active fluid removal has been highlighted in prior studies [8,32], and LDH has been suggested as a diagnostic marker for AMI in ICU patients [17], although laboratory tests often yield non-specific results [[16], [17], [18],^33^]. Our cohort is likely representative of the general ICU population, with a mortality rate in our study is comparable to that reported in the existing literature [2,17,34]. Despite comparable baseline characteristics, the SOFA score differed between groups, reflecting a difference in illness severity that may indicate a higher index of suspicion. We cannot rule out the possibility of overtesting; however, the appropriate rate of true negatives remains debated and depends on the lethality of the disease [35].

We have to note that the inclusion criteria may have been debated because we did not restrict the study to patients who had a laparoscopic confirmation of the diagnosis. However, we have chosen to place the study in the perspective of daily practice where all patients with strong suspicion of AMI do not receive surgical exploration. Our inclusion approach is close to Bourcier et al. [19].

### Limitations

While the indication of bowel resection is well established in cases of NB, the need for surgery in cases of non-transmural AMI is a matter of debate, and takes into account different parameters, including the illness severity of patients [36]. In the most severe patients, bowel resection may be required even in cases of non-transmural injury.

Furthermore, the study only considered the presence of NB at a single time, and did not monitor biomarkers evolution or the potential secondary apparition of NB.

We have to note that the location of NB was not recorded. Even though the surgical literature does not often mix mesenteric ischaemia (small bowel) and colonic ischaemia, the ICU literature often does so, assuming a common pathophysiology, and a common medico-surgical management.

All efforts were made to minimize confounding factors, and we believe that the main known variables that could influence our analysis were included. However, we cannot rule out the possibility of residual bias.

The definition of NB was purely macroscopic, based on surgical observation, which may differ from histological findings. It has been shown that it may be difficult to predict the presence of histology-proven transmural digestive necrosis [37]. A future study may evaluate the correlation between surgical and histology findings.

## Conclusion

In ICU patients suspected of AMI, age, elevated plasma LDH levels, RRT, active fluid removal, and signs of GI injury were predictive of the presence of NB. Surgical exploration to rule out NB should be considered in patients with a high clinical suspicion of AMI, particularly when signs of gastrointestinal injury are present in a context of fluid removal or renal replacement therapy.

## CRediT authorship contribution statement

SA performed statistical analyses, participated in methodology revision and drafted the manuscript.

JA participated in patients’ inclusion and revised statistics.

NA participated in patients’ inclusion and revised the manuscript.

GS participated in statistical analyses and drafted the manuscript.

PM participated to critical revision of the manuscript.

MD participated to the conception of the study and revised the manuscript.

SY participated in the conception of the study and patients' inclusion.

LF participated in patients’ inclusion and revised the manuscript.

YC participated in the conception of the study, patients’ inclusion, and revision of the manuscript.

LRP participated to data collection, patients’ inclusion, revised the manuscript.

PM participated to conception of methodology and critical revision of manuscript.

PA conceived the study, collected data, drafted the manuscript.

## Consent for publication

Not applicable.

## Ethics approval and consent to participate

The study received the ethical approval from an independent ethical committee (CERAR:IRB 00010254 - 2016 – 085). The patients or their next of kin were informed of the study.

## Funding

No funding was received for the conception or realization of this study.

## Availability of data and materials

The datasets used and/or analysed during the current study are available from the corresponding author on reasonable request.

## Declaration of competing interest

The authors declare that they have no competing interests.
