# Supplementary material for: The challenging diagnosis of ICU-related Mesenteric Ischaemia: a prospective, observational, multicentre cohort
Source: Ann Intensive Care. 2026 Jan 21;16:100028. doi: 10.1016/j.aicoj.2026.100028 (PMC12934417; doi:10.1016/j.aicoj.2026.100028)
Supplement: Supplementary file 1 [file mmc1.doc]

| Table S1. Clinical context of high suspicion for AMI | |
| --- | --- |
|  | All patients  202 |
| Unexplained worsening or new-onset organ dysfunction with GI injury, n(%) | 139 (69) |
| Unexplained worsening or new-onset organ dysfunction without GI injury, n(%) | 45 (22) |
| GI injury without unexplained organ dysfunction, n(%) | 18 (9) |
| Abbreviations: AMI = Acute Mesenteric Ischemia; GI = gastro-intestinal injury | |

| Table S2. Diagnostic procedures and results according to the presence of necrotic bowel | | |
| --- | --- | --- |
| Diagnostic test | No NB  128 | NB  74 |
| Contrast enhanced CT (n=129) | n= 86 | n = 43 |
| Ischaemia compatible | 15 (17) | 22 (51) |
| Endoscopy (n=63) | n =47 | n = 16 |
| Moderate ischaemia to necrosis | 14 (30) | 4 (25) |
| Abbreviations: NB = Necrotic Bowel ; CT = Computed Tomography | | |

| Table S3. Patients with surgical exploration – baseline characteristics (patients with surgery) | | | | |
| --- | --- | --- | --- | --- |
| Variables | | No NB  66 (47%) | NB  74 (53%) | p-value |
| Age (years) | | 62 [53;68] | 67 [59;75] | 0.005 |
| Gender (female) | | 12 (18) | 21 (29) | 0.143 |
| Hypertension | | 38 (58) | 53 (72) | 0.082 |
| Type 2 diabetes mellitus | | 18 (27) | 12 (16) | 0.111 |
| Insulin-treated diabetes mellitus | | 5 (8) | 5 (7) | 0.831 |
| Active or past-history of smoking | | 33 (50) | 39 (53) | 0.749 |
| Dyslipidemia | | 21 (32) | 28 (38) | 0.456 |
| CKD | | 12 (18) | 19 (26) | 0.286 |
| Chronic hemodialysis | | 9 (7) | 7 (10) | 0.522 |
| Vascular artery disease | | 36 (55) | 48 (65) | 0.213 |
| - CAD | | 21 (32) | 13 (18) |  |
| - Peripheral AD | | 24 (36) | 38 (52) |  |
| Antiplatelets treatment | | 40 (61) | 39 (53) | 0.346 |
| Statins | | 32 (49) | 38 (51) | 0.735 |
| Beta blockers | | 26 (39) | 27 (37) | 0.723 |
| Calcium inhibitors | | 17 (26) | 11 (15) | 0.106 |
| ACEI or ARB | | 18 (28) | 29 (40) | 0.136 |
| AF (permanent or paroxysmal) | | 21 (32) | 14 (19) | 0.078 |
| Abbreviations: NB = Necrotic Bowel; CKD – Chronic Kidney Disease; CAD = Coronary Artery Disease; SOFA – Sequential Organ Failure Assessment ACEI – Angiotensin Converting Enzyme Inhibitor; ARB – Angiotensin II Receptor Blocker, AF – Atrial Fibrillation | | | | |
|  |  | | | |

| Table S4. Characteristics at ICU admission and prior to AMI suspicion (patients with surgery) | | | |
| --- | --- | --- | --- |
| Variables | No NB  66 (47%) | NB  74 (53%) | p-value |
| Type of hospitalization in ICU |  |  | 1 |
| Medical | 20 (30) | 22 (30) |  |
| Surgical | 47 (70) | 52 (79) |  |
| SOFA upon admission | 9 ± 4 | 10 ± 4 | 0.512 |
| Aortic surgery | 19 (29) | 21 (28) | 0.957 |
| Active fluid removal within the previous 72 hours | 4 (6) | 19 (26) | 0.004 |
| Need for catecholamines within the previous 72 hours | 37 (56) | 42 (57) | 0.934 |
| Abbreviations: NB = Necrotic bowel; ICU = Intensive Care Unit; SOFA = Sequential Organ Failure Assesment; RRT = Renal Replacement Therapy | | | |

| Table S5. Patients’ characteristics at the time of mesenteric ischemia suspicion (patients with surgery) | | | |
| --- | --- | --- | --- |
| Variables | No NB  66 (47) | NB  74 (53) | p-value |
| GI failure | 43 (65) | 65 (88) | 0.001 |
| Organ dyfunction | 63 (96) | 64 (87) | 0.084 |
| Respiratory dysfunction | 36 (56) | 40 (54) | 0.796 |
| Neurological | 16 (24) | 13 (18) | 0.181 |
| New-onset renal failure | 52 (80) | 57 (77) | 0.979 |
| De novo RRT | 31 (46) | 38 (51) | 0.494 |
| Need for catecholamines | 55 (85) | 58 (78) | 0.294 |
| Art. lact D1 (mmol/L) | 3.9 [1.9-9.7] | 3.4 [2-6.6] | 0.326 |
| Arterial pH | 7.26 [7.1-7.38] | 7.25 [7.17-7.37] | 0.619 |
| Alkaline reserve (mmol/L) | 18 [13-21] | 18 [14-21] | 0.966 |
| Creatin Kinase (IU/L) | 550 [152-2043] | 442 [167-3460] | 0.986 |
| Bilirubin (µmol/L) | 14 [10-27] | 14 [10-27] | 0.491 |
| LDH (IU/L) | 519 [277-1113] | 672 [425-1426] | 0.052 |
| - Log (LDH) | 2.72 [2.44-3.05] | 2.84 [2.63-3.18] | 0.052 |
| AST (IU/L) | 127 [50-394] | 109 [52-656] | 0.583 |
| ALT(IU/L) | 53 [27-217] | 73 [31-342] | 0.244 |
| PTap (%) | 62 ± 24 | 58 ± 21 | 0.322 |
| Creatinin (µmol/L) | 167 [120-263] | 200 [134-329] | 0.125 |
| Alkaline phosphatase | 94 [58-182] | 75 [56-111] | 0.050 |
| Hemoglobin (g/dL) | 90 [77-110] | 105 [88-160] | 0.005 |
| WBC (G/L) | 14.8 [10.4-20.6] | 13.0 [8.8-20.2] | 0.335 |
| Platelets (G/L) | 166 [75-263] | 164 [95-235] | 0.893 |
| Abnormal temperature* | 25 (43) | 29 (48) | 0.638 |
| SOFA suspicion | 10 [6-13] | 12 [8-14] | 0.179 |
| Mottled skin | 34 (51) | 41 (55) | 0.225 |
| SAP lowest (mmHg) | 80 [70-88] | 80 [69-92] | 0.280 |
| Abbreviations: NB = Necrotic Bowel; GI = Gastrointestinal; RRT = Renal Replacement Therapy; Art.lact D1 = Total arterial lactate at Day 1 of suspicion; LDH = Lactate Dehydrogenase; AST= Aspartate Aminotransferase; ALT = Alanine Aminotransferase; PTap = Prothrombin time activity percentage; WBC = White Blood Cells; SOFA = Sequential Organ Failure Assessment; SAP = Systolic Arterial Pressure; * = Temperature < 36°C, or >38.5°C. | | | |

| Table S6 . Multivariable logistic regression analysis with NB as dependent variable (patients with surgery) | | | |
| --- | --- | --- | --- |
| Variable | Estimate | OR (95% Confidence Interval) | p |
| Age (1 year) | 0.051 | 1.052 (1.017-1.09) | 0.004 |
| Signs of GI injury (yes vs no) | 0.999 | 2.715 (1.087-6.783) | 0.028 |
| Active fluid removal (yes vs no) | 1.626 | 5.086 (1.615-16.015) | 0.023 |
| Hemoglobin ( 1 g/dL) | -0.01 | 0.99 (0.96-1.02) | 0.517 |
| Abbreviations: NB = Necrotic Bowel; SOFA = Sequential Organ Failure Assessment; GI = Gastrointestinal. | | | |

| Table S7. Area under the curve of biomarkers (all cohort) | |
| --- | --- |
| Biomarker | Area Under the Curve |
| pH | 0.413 |
| Alkaline reserve | 0.400 |
| Lactate Dehydrogenase | 0.685 |
| Aspartate Aminotransferase | 0.602 |
| Alanine Aminotransferase | 0.600 |
| Prothrombin time activity percentage | 0.395 |
| Creatinin | 0.609 |
